# A Government Investigation of Adulteration

**Published:** 1897-11

**Authors:** 


### A Government Investigation of Adulteration.

Mr. A. J. Wedderburn, a special agent of the United States Department of Agriculture, Division of Chemistry, has issued a circular, dated September 17, 1897, in which he announces that, by direction of Congress, the department is investigating the character and extent of the adulteration of foods and drugs. It is generally believed, he says, that adulteration, sophistication, imitation, and misbranding of foods, drugs, and liquors exist to a very great extent. Many of the States have enacted laws to prevent such practices, and it is very desirable to know how these laws have been enforced, and with what results. As the general public is largely interested in this matter, as it affects health, morals, and legitimate trade, it is thought proper to ask the co-operation of the press in securing accurate information on the subject. The publication of a simple request for information on this subject, to be furnished the paper asking it, or sent direct to the Chemical Division of the Department of Agriculture, will in all probability secure a large amount of valuable data which will materially assist in properly carrying out the work. As no matter can be of more importance to the people of the United States than that of the extent and character of the adulteration of foods and drugs sold to them, he asks for co-operation in the work as herein indicated. "Please state," he says, "that the department simply desires a concise statement of facts which can be fully sustained if necessary, and not theories."

The questions that Mr. Wedderburn wishes to have answered are the following:

(1) Do you know of any new adulterant? If yes, state what and how used.

(2) Would a national food and drug law assist in preventing adulteration?

(3) Would uniform food, drug, and pharmaceutical laws tend to promote efficiency and purity?

(4) Please suggest what would best promote the interests of consumers and legitimate manufacturers and dealers.

(5) What is your opinion as to the extent of damage done legitimate business by imitation of brands, packages, etc.?

(6) To what extent do sophistication, misbranding, and injurious adulteration exist?

(7) Have State laws aided in preventing adulteration? To what extent?

(8) Would a national law assist State officials in properly executing the local laws?

(9) Have adulteration, sophistication, and misbranding increased or decreased?

